# Successful Treatment of Peripheral T-cell Lymphoma Not Otherwise Specified in Bilateral Subconjunctiva: A Case Report

**DOI:** 10.7759/cureus.100285

**Published:** 2025-12-28

**Authors:** Haruka Ota, Koji Arihiro, Keichiro Mihara, Tai-ichiro Chikama

**Affiliations:** 1 Ophthalmology, Kure Saiseikai Hospital, Hiroshima, JPN; 2 Anatomical Pathology, Hiroshima University, Hiroshima, JPN; 3 Cell and Gene Therapy, Fujita Health University, Toyoake, JPN; 4 Ophthalmology and Visual Sciences, Graduate School of Biomedical and Health Sciences, Hiroshima University, Hiroshima, JPN

**Keywords:** bilateral, chop, conjunctival, lymphoma, ptcl-nos

## Abstract

Peripheral T-cell lymphoma not otherwise specified (PTCL-NOS) typically presents in lymph nodes with advanced-stage disease. Ocular adnexal involvement, particularly bilateral subconjunctival presentation, is exceptionally rare. We describe a 39-year-old woman who presented with progressive bilateral conjunctival masses despite one year of topical corticosteroid treatment. Examination revealed well-demarcated pink subconjunctival nodules in both eyes. Following surgical excision, histopathological and immunophenotypic analyses confirmed PTCL-NOS with CD3+/CD4+ T-cell infiltration. Molecular studies detected clonal T-cell receptor gene rearrangement in both tumor tissue and bone marrow, indicating stage II disease. The patient achieved complete remission with CHOP (cyclophosphamide, doxorubicin, vincristine, and prednisolone) chemotherapy and remains disease-free after three years. To our knowledge, this represents the first reported case of primary bilateral subconjunctival PTCL-NOS successfully managed with systemic chemotherapy, highlighting the importance of comprehensive evaluation and aggressive treatment in this unusual presentation.

## Introduction

We describe a 39-year-old woman who presented with progressive bilateral conjunctival masses despite one year of topical corticosteroid treatment. Furthermore, we describe the successful treatment outcome with CHOP (cyclophosphamide, doxorubicin, vincristine, and prednisolone) chemotherapy.

Peripheral T-cell lymphoma not otherwise specified (PTCL-NOS) often presents in lymph nodes. The majority of patients with PTCL-NOS have advanced disease at diagnosis and a poor prognosis. Common extranodal sites of involvement, observed in at least 10% of patients, include the spleen, bone marrow, liver, and skin [1].

PTCL-NOS is a diagnosis of exclusion and thus represents a vastly heterogeneous group of diseases. According to the WHO Classification of Tumors of Hematopoietic and Lymphoid Tissues (5th edition), PTCL-NOS accounts for approximately 25%-30% of all peripheral T-cell lymphomas and typically presents with advanced-stage disease [2]. The diagnosis of PTCL-NOS requires careful exclusion of other specific T-cell lymphoma entities through a combination of morphological features and immunophenotypic profile [3].

Ocular adnexal lymphomas are primary extranodal lymphomas, with most being of the marginal zone mucosa-associated lymphoid tissue (MALT) type. Primary subconjunctival PTCL-NOS appears to be exceptionally rare in the literature. To our knowledge, this appears to be a rare presentation of PTCL-NOS with bilateral conjunctival involvement as the primary manifestation, achieving persistent complete remission (CR) with CHOP therapy.

## Case presentation

History

**Figure 1:**
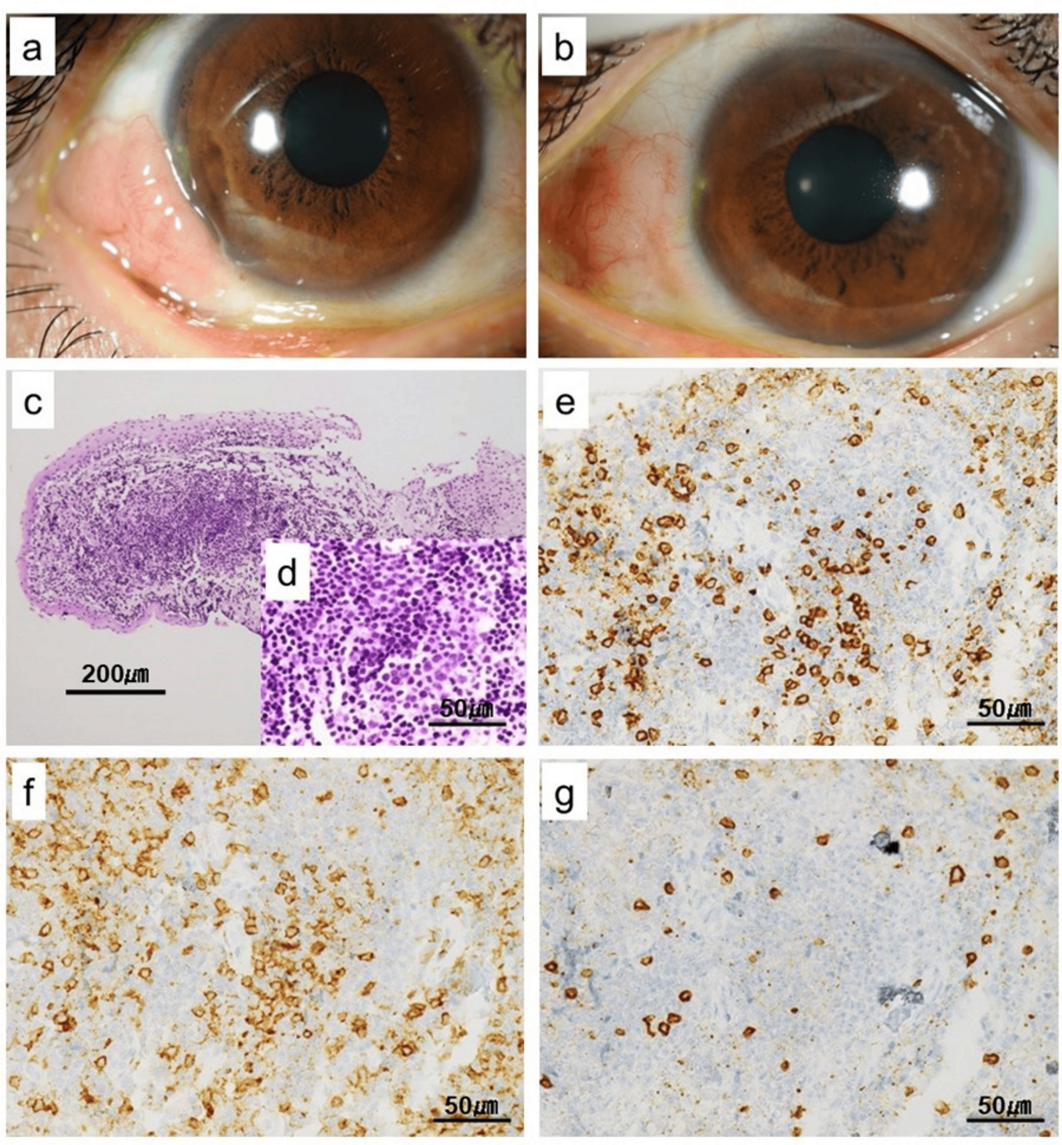
The tumors were seen in subconjunctival lesions of the temporal and the nasal side of the right eye and the inferonasal side of the left eye, which were relatively hard and well-bounded pink nodules with smooth surfaces ((a) right eye; (b) left eye). (c, d) Morphology of cells in the conjunctival tumor resected, with hematoxylin-eosin staining. Monomorphic small-to-medium-sized lymphocytes with slightly irregular nuclei diffusely infiltrated throughout the corneal epithelial lesions. (e-g) Immunohistochemical results of abnormal lymphocytes in the resected mass. They were positive for CD3 and CD4. They were negative for CD8 ((e) CD3, (f) CD4, and (g) CD8)

A 39-year-old woman was referred to our hospital for bilateral conjunctival masses that had enlarged despite medical treatment with a therapeutic eye drop containing steroids for a year. She had a medical history of depression. Visual acuity was 20/16 in the right eye and 20/40 in the left eye. Intraocular pressures were 13 mmHg OD and 16 mmHg OS. The tumors were seen in subconjunctival lesions of the temporal and the nasal side of the right eye and the inferonasal side of the left eye, which were relatively hard and well-bounded pink nodules with smooth surfaces (Figure 1).

Examination

Superficial punctate keratopathy was present on the peripheral corneas adjacent to the tumors. The optic media were clear, and the optic nerve head and retina showed no abnormalities. Peripheral blood analysis showed that aspartate aminotransferase (AST) (13 U/L; normal range: 10-40 U/L) and blood urea nitrogen (BUN) (7.9 mg/dL; normal range: 8-20 mg/dL) were slightly elevated. The serum lactate dehydrogenase value (125 U/L; normal range: 120-220 U/L) and soluble interleukin-2 receptor (442 U/mL; normal range: 122-496 U/mL) were within normal range. Positron-emission tomography/computed tomography demonstrated that there was no swollen lymph node, and 18F-fluorodeoxyglucose (FDG) was not significantly incorporated in any lesion including the conjunctivae.

The initial appearance of the tumor mass suggested a diagnosis of lymphoma, and then, the nodular subconjunctival tumors in both eyes were removed. Pathological results demonstrated that T-cells featuring slightly irregular nuclei, which were positive for CD3 and CD4 and negative for CD8 and CD20, diffusely proliferated, which was consistent with flow cytometric results on the tumor excised. Rearrangement of the T-cell receptor (TCR)-d gene was clonally detected by a PCR-based clonality assay, albeit without rearrangement of the immunoglobulin heavy chain gene. Bone marrow examination showed no evidence of involvement of lymphoma cells with flow cytometry and morphological analysis under light microscopy. However, rearrangement of the TCR-d gene was detected in the bone marrow, suggestive of molecular involvement of lymphoma cells. Clinical staging according to the Ann Arbor classification revealed stage II disease, defined by involvement of conjunctival tissue (extranodal site) with molecular evidence of bone marrow involvement without morphological infiltration.

Management

We diagnosed her with PTCL-NOS with clinical stage II. The diagnosis of PTCL-NOS was established according to WHO criteria, which require the exclusion of other specific T-cell lymphoma subtypes through comprehensive morphological, immunophenotypic, and molecular analysis [2]. Diagnostic workup followed established criteria for PTCL-NOS, requiring demonstration of a clonal T-cell population with CD3+ phenotype, absence of specific markers that would classify the tumor into other T-cell lymphoma categories, and exclusion of anaplastic large cell lymphoma, angioimmunoblastic T-cell lymphoma, and other defined entities [3]. She received CHOP chemotherapy, leading to CR, and thereafter, there has been no evidence of local or systemic recurrence for three years.

## Discussion

We report a case of PTCL-NOS in bilateral conjunctiva as an initial presentation and then successful treatment with CHOP chemotherapy. The differential diagnosis of bilateral conjunctival masses includes reactive lymphoid hyperplasia, MALT lymphoma, diffuse large B-cell lymphoma (DLBCL), and other T-cell lymphoproliferative disorders. The combination of clinical presentation, histomorphology, immunophenotyping, and molecular studies was essential for accurate diagnosis.

The diagnosis followed WHO classification guidelines, systematically excluding other defined T-cell lymphoma entities based on morphologic, immunophenotypic, and molecular findings. The conjunctiva contains specialized lymphoid tissue, including the MALT system [4], and conjunctival lymphomas constitute 25% of all ocular adnexal lymphomas.

Large epidemiological studies have provided valuable insights into ocular adnexal lymphomas. Vest et al. conducted a population-based study of 1,168 patients with ocular adnexal lymphomas in America; the most frequent lymphoma subtypes were extranodal marginal zone B-cell lymphoma (EMZL) (n = 688, 59%), follicular lymphoma (FL) (n = 150, 13%), DLBCL (n = 131, 11%), and mantle cell lymphoma (MCL) (n = 89, 8%) [5]. This emphasizes the exceptional rarity of our case, as T-cell lymphomas involving the conjunctiva are extraordinarily uncommon.

Conjunctival lymphomas are most commonly EMZL and FL, both of which are generally low-grade. EMZL, previously known as MALT lymphoma, constitutes approximately 80% of conjunctival B-cell non-Hodgkin lymphomas (NHLs). Conjunctival T-cell lymphoma is a very rare entity.

Conjunctival lymphoma classically presents as a pink, salmon-colored subconjunctival mass [6] and presents with chronic follicular conjunctivitis [7]. Bilateral lesions account for 10%-15% of cases of conjunctival lymphoma overall [8,9].

Therefore, PTCL-NOS is extremely rare in ocular adnexal lymphoma. In addition, conjunctival T-cell lymphoma is a very rare entity.

The management of conjunctival lymphoma is varied and tailored to the type of lymphoma. Treatment options discussed include radiation, chemotherapy, immunotherapy, antibiotic therapy, and combination regimens.

Treatment selection was guided by the National Comprehensive Cancer Network (NCCN) Guidelines for T-Cell Lymphomas, which recommend CHOP or CHOP-like regimens as first-line therapy for peripheral T-cell lymphomas, including PTCL-NOS [10]. The guidelines emphasize the importance of systemic therapy over local treatment for patients with advanced-stage disease or evidence of bone marrow involvement [10]. Although local radiotherapy is considered appropriate for early-stage ocular adnexal lymphomas, the NCCN guidelines support the use of systemic chemotherapy in cases with molecular evidence of dissemination, as observed in our patient [10].

T-cell NHLs are uncommon malignancies. Because of the rarity of these disorders and the lack of well-designed clinical trials, the treatment of peripheral T-cell NHLs is often challenging.

PTCL-NOS is a clinically aggressive disease, with a poor response to therapy and an approximate five-year overall survival of 30% [11]. Weisenburger et al. reported the clinical features of the 340 patients with PTCL-NOS; the majority (69%) presented with advanced-stage disease [12]. At the close of the study, 68% of the patients had died, only 9% were in remission at the time of death, and the five-year overall survival was 32% [12].

Coupland et al. reported a case of PTCL-NOS of subconjunctival masses in the right eye, symblepharon, and successful treatment with surgical excision and Rx (Table 1) [13]. The patient had no recurrence after five years of treatment (Table 1) [13]. And they reported that treatment of CHOP chemotherapy in two cases of PTCL-NOS resulted in death [13]. Mito et al. reported a case of PTCL-NOS of ulcers in the left eyelid skin, treated with irradiation (Table 1) [14]. The patient had no recurrence at six months [14]. Iluta et al. reported a case of PTCL-NOS of a large tumoral mass in the right frontal area, with involvement of the right eyelid and the ocular globe, treated successfully with COP (cyclophosphamide, Oncovin (vincristine), and prednisone) plus etoposide combination chemotherapy (Table 1) [15]. The patient died of acute respiratory distress syndrome after completion of chemotherapy (Table 1) [15].

**Table 1:** Clinical information of the ocular PTCL of 3 cases COP: cyclophosphamide, Oncovin (vincristine), and prednisone; PTCL: peripheral T-cell lymphoma

Study	Age	Symptoms	Immunophenotypic analyses	Stage	Treatment	Outcome
Coupland et al. [13]	76	Subconjunctival masses in the right eye	Positive for CD45, CD3, and CD8	IE	Surgical excision plus Rx	Alive
Mito et al. [14]	27	Left lower eyelid	Positive for CD4, CD3, and CD5; negative for CD8, CD20, and CD56	I	Rx	Alive
lluta et al. [15]	72	A large tumoral mass in the right frontal area, with involvement of the right eyelid and the ocular globe	Positive for CD3, CD4, CD8, CD20, and CD68; negative for CD30	IV	Etoposide plus COP	Death

The choice of systemic chemotherapy over local radiotherapy was based on several factors: (1) bilateral involvement suggesting systemic disease, (2) molecular evidence of bone marrow involvement despite negative morphology, (3) the aggressive nature of PTCL-NOS with poor prognosis, and (4) current treatment guidelines recommending systemic therapy for advanced-stage disease. Prognostic factors in PTCL-NOS include age, performance status, stage, and bone marrow involvement. Our patient's young age and achievement of CR with standard CHOP therapy represent a favorable outcome, contrasting with the generally poor prognosis of PTCL-NOS.

The International T-Cell Lymphoma Project, which analyzed 1,314 cases of PTCL from 22 institutions worldwide, confirmed the heterogeneous nature and poor overall survival of PTCL-NOS, with a five-year overall survival of 32% [16]. Our patient's sustained CR represents a favorable outcome within this generally poor prognostic group. Therefore, this successful treatment case is rare.

## Conclusions

Irradiation is an adequate treatment option for patients with ocular adnexal lymphoma. However, as lymphoma cells could be molecularly spread into bone marrow and the prognosis of PTCL-NOS is poor, we chose CHOP chemotherapy, and resultantly, she achieved a sustainable CR. These findings highlight a careful necessity for clinicians to decide to treat patients with a rare case of PTCL-NOS in the conjunctiva. This is a single case report; the study is limited. Broader validation is warranted to validate this result and to generalize the treatment outcome to ocular PTCL-NOS cases.

## References

[1] Weiss J, Reneau J, Wilcox RA (2023). PTCL, NOS: an update on classification, risk-stratification, and treatment. Front Oncol.

[2] Loghavi S, Kanagal-Shamanna R, Khoury JD, Medeiros LJ, Naresh KN, Nejati R, Patnaik MM (2024). Fifth edition of the World Health Classification of Tumors of the Hematopoietic and Lymphoid Tissue: myeloid neoplasms. Mod Pathol.

[3] Swerdlow SH, Campo E, Pileri SA (2016). The 2016 revision of the World Health Organization classification of lymphoid neoplasms. Blood.

[4] Isaacson PG, Du MQ (2004). MALT lymphoma: from morphology to molecules. Nat Rev Cancer.

[5] Vest SD, Coupland SE, Esmaeli B (2023). Specific location of ocular adnexal lymphoma and mortality: an international multicentre retrospective study. Br J Ophthalmol.

[6] Shields CL, Chien JL, Surakiatchanukul T, Sioufi K, Lally SE, Shields JA (2017). Conjunctival tumors: review of clinical features, risks, biomarkers, and outcomes--the 2017 J. Donald M. Gass lecture. Asia Pac J Ophthalmol (Phila).

[7] Meunier J, Lumbroso-Le Rouïc L, Dendale R (2006). Conjunctival low-grade non-Hodgkin's lymphoma: a large single-center study of initial characteristics, natural history and prognostic factors. Leuk Lymphoma.

[8] Seker M, Ozdemir B, Bilici A (2010). Bilateral conjunctival MALT lymphoma mimicking chronic conjunctivitis. Onkologie.

[9] Ferreri AJ, Dolcetti R, Du MQ (2008). Ocular adnexal MALT lymphoma: an intriguing model for antigen-driven lymphomagenesis and microbial-targeted therapy. Ann Oncol.

[10] Horwitz SM, Ansell S, Ai WZ (2022). T-cell lymphomas, version 2.2022, NCCN Clinical Practice Guidelines in Oncology. J Natl Compr Canc Netw.

[11] Mourad N, Mounier N, Brière J (2008). Clinical, biologic, and pathologic features in 157 patients with angioimmunoblastic T-cell lymphoma treated within the Groupe d'Etude des Lymphomes de l'Adulte (GELA) trials. Blood.

[12] Weisenburger DD, Savage KJ, Harris NL (2011). Peripheral T-cell lymphoma, not otherwise specified: a report of 340 cases from the International Peripheral T-cell Lymphoma Project. Blood.

[13] Coupland SE, Foss HD, Assaf C (1999). T-cell and T/natural killer-cell lymphomas involving ocular and ocular adnexal tissues: a clinicopathologic, immunohistochemical, and molecular study of seven cases. Ophthalmology.

[14] Mito H, Kakizaki H, Tsuji H, Ide A, Takeuchi K, Takamura H (2006). Peripheral T-cell lymphoma of the eyelid. Jpn J Ophthalmol.

[15] Iluta S, Termure DA, Petrushev B (2020). Clinical remission in a 72-year-old patient with a massive primary cutaneous peripheral T-cell lymphoma-NOS of the eyelid, following combination chemotherapy with etoposide plus COP. Diagnostics (Basel).

[16] Vose J, Armitage J, Weisenburger D (2008). International peripheral T-cell and natural killer/T-cell lymphoma study: pathology findings and clinical outcomes. J Clin Oncol.

